# NRPB3, the third largest subunit of RNA polymerase II, is essential for stomatal patterning and differentiation in *Arabidopsis*

**DOI:** 10.1242/dev.129098

**Published:** 2016-05-01

**Authors:** Liang Chen, Liping Guan, Pingping Qian, Fan Xu, Zhongliang Wu, Yujun Wu, Kai He, Xiaoping Gou, Jia Li, Suiwen Hou

**Affiliations:** Key Laboratory of Cell Activities and Stress Adaptations, Ministry of Education, School of Life Sciences, Lanzhou University, Lanzhou 730000, People's Republic of China

**Keywords:** Stomata, RNA polymerase II, Patterning, Differentiation, *Arabidopsis*

## Abstract

Stomata are highly specialized epidermal structures that control transpiration and gas exchange between plants and the environment. Signal networks underlying stomatal development have been previously uncovered but much less is known about how signals involved in stomatal development are transmitted to RNA polymerase II (Pol II or RPB), which plays a central role in the transcription of mRNA coding genes. Here, we identify a partial loss-of-function mutation of the third largest subunit of nuclear DNA-dependent Pol II (*NRPB3*) that exhibits an increased number of stomatal lineage cells and paired stomata. Phenotypic and genetic analyses indicated that NRPB3 is not only required for correct stomatal patterning, but is also essential for stomatal differentiation. Protein-protein interaction assays showed that NRPB3 directly interacts with two basic helix-loop-helix (bHLH) transcription factors, FAMA and INDUCER OF CBF EXPRESSION1 (ICE1), indicating that NRPB3 serves as an acceptor for signals from transcription factors involved in stomatal development. Our findings highlight the surprisingly conserved activating mechanisms mediated by the third largest subunit of Pol II in eukaryotes.

## Abstract (precis)

**Summary:** RNA polymerase II subunit NRPB3 interacts with stomatal bHLH transcription factors FAMA and ICE1, connecting the stomatal development pathway to the general transcription machinery.

## INTRODUCTION

Stomata, which consist of paired guard cells, are known to have played crucial roles in the colonization of land by plants. Turgor-driven stomatal movement requires ion and water exchange with neighboring cells and controls transpiration and gas exchange between plants and the environment. To function efficiently, the development of stomata complies with the one-cell-spacing rule, that is, two stomata are separated by at least one non-stomatal cell. In *Arabidopsis*, the stomatal lineage begins with an asymmetric entry division, which takes place in a fraction of protodermal cells known as meristemoid mother cells (MMCs). The division gives rise to two daughter cells with distinct morphologies: a large sister cell known as the stomatal lineage ground cell (SLGC) and a small triangular meristemoid. The meristemoid undergoes asymmetric amplifying division and regenerates an SLGC and a meristemoid that ultimately converts into a guard mother cell (GMC). The GMC divides symmetrically once to form a pair of guard cells (GCs) (Nadeau and Sack, 2002a; Bergmann and Sack, 2007). The SLGCs produced by asymmetric entry and amplifying divisions can either initiate stomatal development by undergoing oriented asymmetric spacing division or terminally differentiate into pavement cells (Geisler et al., 2000).

Several key genes and regulatory networks underlying stomatal development have been uncovered by molecular genetic analyses. Three ERECTA family (ERf) leucine-rich repeat receptor-like kinases [LRR-RLKs; ER, ERECTA-LIKE1 (ERL1) and ERL2], four SOMATIC EMBRYOGENESIS RECEPTOR KINASE (SERK) LRR-RLKs (SERK3/BAK1, SERK2, SERK1 and SERK4) and a leucine-rich repeat receptor-like protein (LRR-RLP) TOO MANY MOUTHS (TMM) have been identified as stomatal development receptors (Nadeau and Sack, 2002b; Shpak et al., 2005; Meng et al., 2015). Regarding their ligands, several small secreted, putative peptides belonging to the EPIDERMAL PATTERNING FACTOR-LIKE (EPFL) family have been discovered. Among these peptides, EPF1, EPF2 and CHALLAH family ligands (EPFL4-EPFL6) are negative regulators of stomatal density (Hara et al., 2007, 2009; Hunt and Gray, 2009; Abrash and Bergmann, 2010; Abrash et al., 2011; Lee et al., 2012; Niwa et al., 2013). By contrast, EPFL9/STOMAGEN positively regulates stomatal density (Hunt et al., 2010; Kondo et al., 2010; Sugano et al., 2010; Lee et al., 2015). A mitogen-activated protein kinase (MAPK) cascade, which consists of a MAPKKK (YODA), four MAPKKs (MKK4/5/7/9) and two MAPKs (MPK3/6), regulates stomatal development downstream of the receptors (Bergmann et al., 2004; Wang et al., 2007; Lampard et al., 2008, 2009). In addition, STOMATAL DENSITY AND DISTRIBUTION1 (SDD1), a putative subtilisin acting upstream of TMM, is also a negative regulator of stomatal density (Berger and Altmann, 2000; von Groll, 2002). All of these genes are stomatal patterning genes, which regulate stomatal development with the correct pattern and proper density (Pillitteri and Torii, 2012).

As intrinsic positive regulators of stomatal differentiation, the closely related basic helix-loop-helix (bHLH) transcription factors, SPEECHLESS (SPCH), MUTE and FAMA control the consecutive cell fate transitions, MMC to meristemoid, meristemoid to GMC and GMC to GCs, respectively (Ohashi-Ito and Bergmann, 2006; MacAlister et al., 2007; Pillitteri et al., 2007). To specify each cell state transition, SPCH, MUTE and FAMA can also form heterodimers with two paralogous bHLH-leucine zipper (bHLH-LZ) transcription factors, INDUCER OF CBF EXPRESSION1 (ICE1) and SCREAM2 (SCRM2) (Kanaoka et al., 2008). In addition, two partially redundant R2R3 MYB transcription factors, FOUR LIPS (FLP) and MYB88, which are independent of FAMA, control stomatal terminal differentiation (GMC to GCs) (Lai et al., 2005; Ohashi-Ito and Bergmann, 2006).

Programs of gene expression, which are induced by developmental signals, lead to the differentiation of a variety of cell types and tissues (Carrera and Treisman, 2008). In eukaryotes, the transcription of mRNA-coding genes, most snRNAs and microRNAs requires the immediate assembly of a pre-initiation complex, including basal transcription factors and RNA polymerase II (Pol II or RPB), at specific DNA sites. Depending on its origin, Pol II consists of 10-14 subunits (Young, 1991). Within the complicated network of interactions among Pol II subunits, the third largest subunit of Pol II (RPB3) plays a central role in Pol II assembly (Acker et al., 1997). In addition to this fundamental function, the role of RPB3 in transcription regulation is still emerging through ongoing research. The bacterial RNA polymerase (RNAP) α subunits that are homologs of RPB3 and RPB11, are involved in RNAP assembly, promoter recognition and transcriptional activation (Ebright and Busby, 1995). The oncoprotein EWS modulates Pol II activity by interacting with RPB3 and RPB5 (Bertolotti et al., 1998). Two special regions required for activator-dependent transcription have been discovered in yeast RPB3, suggesting that RPB3 might be a regulatory target of the transcription activator (Tan et al., 2000). Furthermore, RPB3 participates in tissue-specific transcription and myogenesis by interacting with the transcription factors, myogenin and transcription factor-4 (ATF4) (Corbi et al., 2002; De Angelis et al., 2003), reinforcing the idea that RPB3 might directly accept signals from specific transcription factors.

In *Arabidopsis*, Pol II contains 12 subunits (Ream et al., 2009). Homozygous T-DNA insertion mutants of Pol II genes are lethal (Onodera et al., 2008; Ream et al., 2009), manifesting the importance of Pol II in plant development. Although the basic function of Pol II in mRNA transcription is understood, little is known about its other potential functions in plant cell differentiation. In this study, we report a partial loss-of-function mutant of the third largest subunit of nuclear DNA-dependent Pol II (*NRPB3*). It exhibited an increased number of stomatal lineage cells and stomatal clusters. Similar stomatal phenotypes were observed in a weak allele of the second largest subunit of nuclear DNA-dependent Pol II (*NRPB2*). These results suggested that Pol II plays essential roles in stomatal development. Genetic analysis indicated that *NRPB3* synergistically interacts with stomatal patterning and differentiation regulators. We also found physical associations of NRPB3 with two bHLH transcription factors, FAMA and ICE1. Our study reinforces the idea that mechanisms needed for the differentiation of skeletal muscle cell in animals are also required for stomatal development in plants.

## RESULTS

### Phenotypic analysis and cloning of *nrpb3-1*

**Fig. 1.**
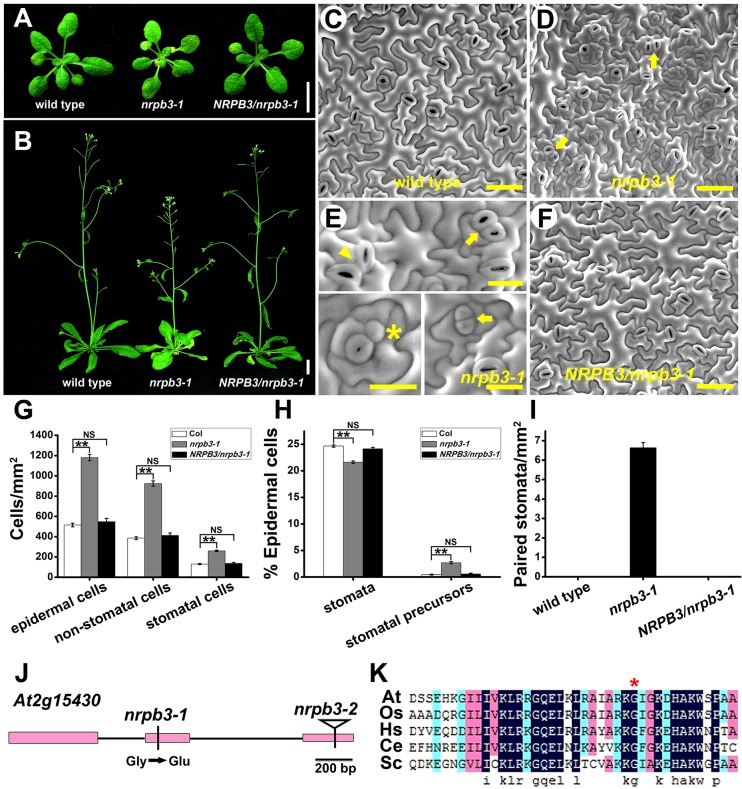
**Isolation of an *nrpb3* mutant.** (A) Two-week-old seedlings of wild type, *nrpb3-1* and *NRPB3*/*nrpb3-1*. (B) Five-week-old plants of wild-type, *nrpb3-1* and *NRPB3*/*nrpb3-1*. (C-F) SEM images of the abaxial epidermis of the seventh fully expanded rosette leaf of wild type (C), *nrpb3-1* (D,E) and *NRPB3*/*nrpb3-1* (F). Arrow, parallel-aligned stomata; arrowhead, none parallel-aligned stomata; asterisk, GMC. (G) Densities of epidermal cells, stomatal cells (meristemoids, GMCs and stomata) and non-stomatal cells on the abaxial epidermis of the seventh mature leaves. (H) The proportion of stomata and stomatal precursors (meristemoids and GMCs) on the abaxial epidermis of the seventh mature leaves. (I) Densities of paired stomata on the abaxial epidermis of the seventh mature leaves. (J) The *Arabidopsis NRPB3* locus. Boxes, exons; lines, introns. The *nrpb3-1* missense allele is indicated by a vertical line and the *nrpb3-2* insertion allele is indicated by a triangle. (K) Partial amino acid sequence of RPB3 in various species. At, *Arabidopsis thaliana*; Os, *Oryza sativa*; Hs, *Homo sapiens*; Ce, *Caenorhabditis elegans*; Sc, *Saccharomyces cerevisiae*; asterisk, mutation site in *nrpb3-1*. Error bars indicate s.e.m.; NS, not significant; **P<0.01 by Student's *t*-test. *n*=30 per genotype. Scale bars: 1 cm in A,B; 50 µm in C,D,F; 20 µm in E.

To identify new genes involved in stomatal development, we isolated a mutant with increased stomatal density and paired stomata in an ethyl methanesulfonate mutagenesis screen. The mutant displayed deficient developmental phenotypes, such as etiolation, late flowering and dwarfness (Fig. 1A,B). Its fully expanded rosette leaves were smaller than those of the wild type (see Fig. S1), suggesting that the mutation resulted in a defect in leaf expansion. Epidermal cell density in the abaxial epidermis of the mutant leaves was increased and further statistical analysis showed that both the number of stomatal cells (meristemoids, GMCs and stomata) and the number of non-stomatal cells were much higher in the mutants than in the wild type (Fig. 1C,D,G). These results suggested that the mutated gene is broadly involved in restraining cell divisions in the entire epidermis. In addition, compared with the wild type, the proportion of stomata was decreased, whereas the proportion of stomatal precursors (meristemoids and GMCs) was greatly increased in both the true leaves and cotyledons of the mutant (Fig. 1H and see Fig. S2A,B,D). By examining the time course of stomatal differentiation in germinating cotyledons with the stomatal lineage reporter *TMM_pro_::TMM-GFP* (Nadeau and Sack, 2002b), we found that the mutant cotyledons produced larger stomatal lineage cell clusters and more stomatal lineage cells compared with the wild type at each developmental stage (see Fig. S3). Furthermore, the number of paired stomata was significantly higher in the mutant (Fig. 1I). Approximately 63% (*n*=144) of the paired stomata were two non-parallel aligned stomata and the remaining were parallel-aligned stomata (Fig. 1E).

To identify the mutated gene, map-based cloning was performed and a G-to-A substitution at nucleotide position 769 of *At2g15430*, which encodes NRPB3, was found (Fig. 1J). This mutation resulted in the conversion of a highly conserved glycine in RPB3 proteins from plants, animals and yeast to glutamic acid (Fig. 1K). We named this mutant *nrpb3-1*.

The *NRPB3* genomic sequence driven by its own 1.6 kb promoter (*NRPB3_pro_::NRPB3-GFP*) was introduced into *nrpb3-1* (*NRPB3/nrpb3-1*) and it completely rescued the deficient phenotypes (Fig. 1A-I). Its T-DNA insertion line (*nrpb3-2*, SALK_008220) (Fig. 1J) was lethal. Arrested ovules and aborted seeds were observed in the siliques of selfed *nrpb3-2* heterozygous plants (see Fig. S4A). Developmental defects similar to *nrpb3-1* were also observed in *nrpb3-1/nrpb3-2* plants (see Fig. S4B-G). Therefore, we conclude that *nrpb3-1* is a partial loss-of-function allele of *NRPB3*. We overexpressed *NRPB3* but no visible deficient phenotypes were observed (see Fig. S5), suggesting that NRPB3 functions as part of the core of Pol II rather than an individual regulator in plant development.

### Downregulation of *NRPB3* dramatically disrupts proper stomatal patterning and differentiation

**Fig. 2.**
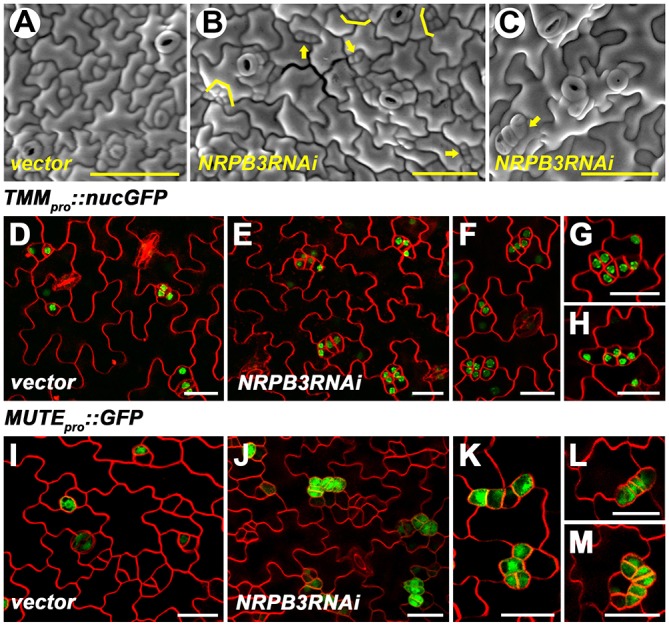
***GVG-NRPB3RNAi* transgenic plants display severe stomatal development defects.** (A-C) SEM images of the abaxial epidermis of the sixth immature rosette leaf of vector control (A) and *GVG-NRPB3RNAi* (B,C) transgenic plants. Arrows indicate caterpillar-like structures in B and clustered stomata in C; bracket, meristemoid-like cell cluster. (D-H) The expression of *TMM_pro_::nucGFP* in the abaxial epidermis of the sixth immature rosette leaf of vector control (D) and *GVG-NRPB3RNAi* (E-H) transgenic plants. (F-H) Close-up of clusters of small, highly divided meristemoid-like cells expressing *TMM_pro_::nucGFP* in *GVG-NRPB3RNAi* transgenic plants. (I-M) The expression of *MUTE_pro_::GFP* in the abaxial epidermis of the sixth immature rosette leaf of vector control (I) and *GVG-NRPB3RNAi* (J-M) transgenic plants. (K-M) Close-up of multiple adjacent cells or caterpillar-like structures expressing *MUTE_pro_::GFP* in *GVG-NRPB3RNAi* transgenic plants. Scale bars: 50 µm in A-C; 20 µm in D-M.

To further elucidate the role of *NRPB3* in stomatal development, we generated plants with dexamethasone (Dex)-inducible RNAi gene silencing of *NRPB3*. Two-week-old T1 transgenic plants were continuously treated with Dex for 10 days and ∼40% (*n*=167) of the *GVG-NRPB3RNAi* transgenic plants displayed stomatal developmental defects including caterpillar-like structures similar to those of *fama*, meristemoid-like cell clusters and paired stomata (Fig. 2A-C). In addition, we constructed two specific *amiR-NRPB3* lines, *amiR-NRPB3-1* and *amiR-NRPB3-2* (see Fig. S6A,B). In *amiR-NRPB3-1* T1 transgenics, 42/48 plants exhibited severe growth defects and clusters of meristemoid-like cells and stomata (see Fig. S6C,E-G). Statistical analysis revealed that the proportion of stomatal precursors dramatically increased in the abaxial epidermis of *amiR-NRPB3-1* cotyledons at 6 days after germination (dag) (see Fig. S2A,C,D). The expression level of *NRPB3* in *amiR-NRPB3-1* decreased to ∼20% of that in the wild type (see Fig. S6D). However, we could barely recover transformants from two independent transformations when *amiR-NRPB3-2* was transformed into wild-type plants.

To characterize the clustered meristemoid-like cells in leaves of *GVG-NRPB3RNAi* transgenic plants, we investigated the expression patterns of the stomatal cell-specific markers *TMM*, which marks stomatal lineage cells (Nadeau and Sack, 2002b) and *MUTE*, which marks late meristemoids, GMCs and immature GCs (Pillitteri et al., 2007). In *GVG-NRPB3RNAi* plants transformed with *TMM_pro_::nucGFP*, clusters of small, highly divided meristemoid-like cells exhibited strong GFP signals (Fig. 2D-H), suggesting that downregulation of *NRPB3* leads to a large increase in disorganized stomatal lineage divisions. In *GVG-NRPB3RNAi* plants transformed with *MUTE_pro_::GFP*, fluorescence could be detected in clusters of immature stomata and multiple adjacent cells, which likely eventually formed stomatal clusters (Fig. 2I-K). Importantly, caterpillar-like structures similar to those of *fama* also expressed *MUTE_pro_::GFP* (Fig. 2K-M). These findings suggested that *NRPB3* is required for limiting stomatal lineage cell divisions.

### Expression pattern and subcellular localization of NRPB3

**Fig. 3.**
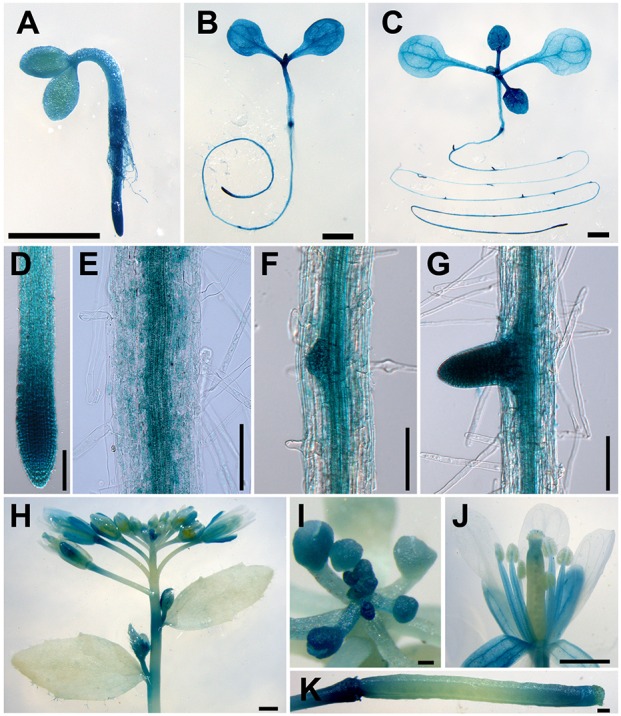
**Expression patterns of *NRPB3*.** (A-C) *NRPB3_pro_::NRPB3-GUS* expression in 1 dag (A), 4 dag (B) and 8 dag (C) seedlings. (D-G) Stronger *NRPB3_pro_::NRPB3-GUS* expression in the meristematic and elongation zone of root tip (D), stele (E), lateral root primordium (F) and newly formed lateral root (G). (H-K) GUS activity in inflorescences and axillaries. (H) *NRPB3_pro_::NRPB3-GUS* expression in the whole developing inflorescence. (I) Inflorescence apex that shows strong GUS expression. (J) GUS activity in a single flower. (K) GUS activity in a newly formed silique. Scale bars: 1 mm in A-C,J; 0.5 mm in H; 100 µm in D-G,I; 200 µm in K.

Histochemical expression pattern analysis showed that *NRPB3* was expressed in almost all tissues. In seedlings, strong *NRPB3* expression was observed in both the shoot and root, and high GUS activity was detected in the shoot apex, root tip, stele, lateral root primordium and newly formed lateral root (Fig. 3A-G). In developing inflorescences, strong staining was present in immature axillaries, the inflorescent apex, and the silique apex and base (Fig. 3H-K).

**Fig. 4.**
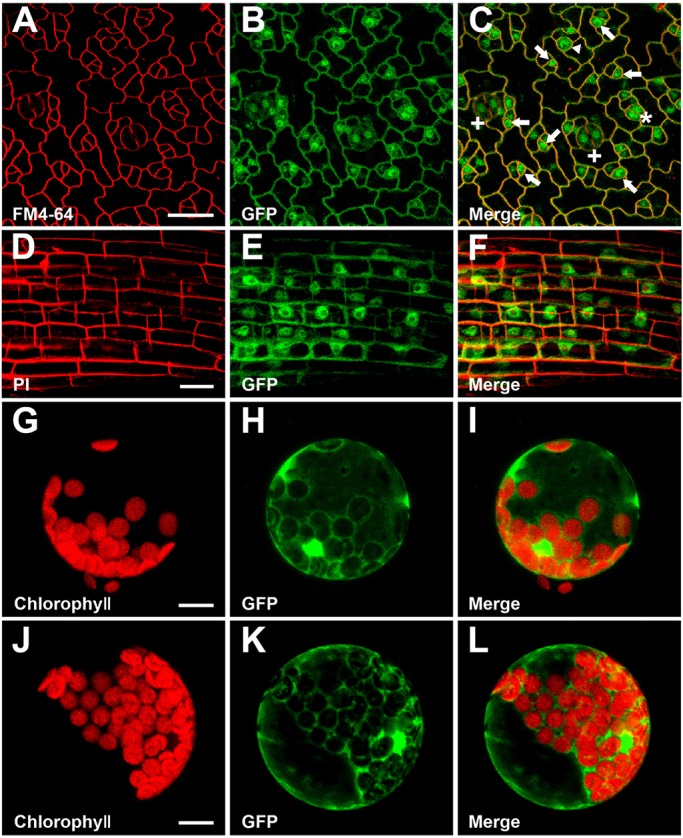
**The expression of *NRPB3* in stomatal lineage cells and the subcellular localization of NRPB3.** (A-C) *NRPB3_pro_::NRPB3-GFP* expression in the leaf epidermis. Arrow, meristemoid; arrowhead, GMC; asterisk, immature stomata; plus, mature stomata. (D-F) Localization of NRPB3-GFP driven by the native *NRPB3* promoter in cells of the root elongation zone. (G-I) *Arabidopsis* protoplasts transiently expressing GFP. (J-L) *Arabidopsis* protoplasts transiently expressing NRPB3-GFP. Scale bars: 20 µm in A-F; 10 µm in G-L.

At the cellular level, *NRPB3* was broadly expressed in the leaf epidermal cells (Fig. 4A-C). In the cells of the root elongation zone, we observed NRPB3-GFP in the nucleus (Fig. 4D-F). Transient expression of NRPB3-GFP in *Arabidopsis* protoplasts indicated that it localized to the cytoplasm as well as the nucleus (Fig. 4G-L).

### NRPB3 is essential for the proper expression of stomatal development genes

The fact that NRPB3 was a key subunit of Pol II led us to investigate the expression levels of genes for stomatal development in *nrpb3-1*. Except for *EPF2*, negative stomatal patterning regulators *TMM*, *ER*, *EPF1*, *YODA* and *SDD1* were significantly down regulated (see Fig. S7B), consistent with the deficient stomatal phenotypes observed in *nrpb3-1*. Regarding the stomatal-promoting genes, *SPCH* and *MUTE* transcripts were abundant in the mutants (see Fig. S7B), consistent with the increased number of stomatal lineage cells in *nrpb3-1*. To confirm the RT-PCR results, *TMM_pro_::TMM-GFP* and *SPCH_pro_::nucGFP* were crossed to *nrpb3-1*, and the fluorescence intensity at the base of the fifth rosette leaf was compared between the wild type and *nrpb3-1* under the same conditions. Weaker *TMM* expression and stronger *SPCH* expression were observed in *nrpb3-1* (see Fig. S7C-F). The relative expression of these genes was also detected in *GVG-NRPB3RNAi* and *amiR-NRPB3-1* transgenic plants and the results were similar to those of *nrpb3-1* (see Fig. S7G,H). These results indicated that NRPB3 is essential for the proper expression of stomatal development genes.

### *NRPB3* interacts synergistically with stomatal patterning genes

**Fig. 5.**
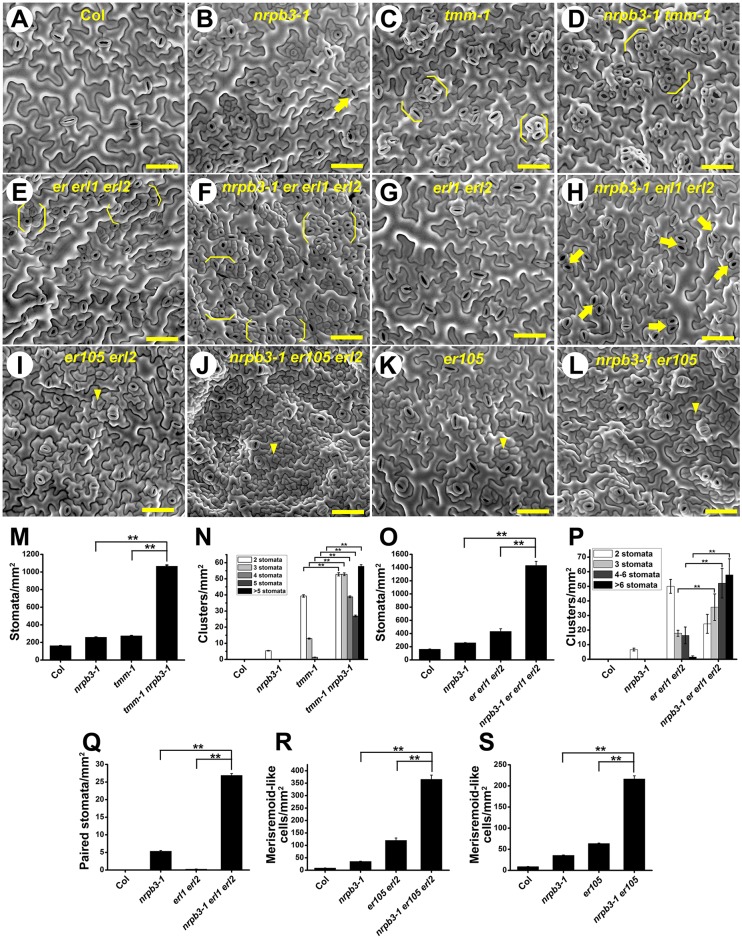
**Genetic interaction analysis between *NRPB3* and the receptors of stomatal development.** (A-L) SEM images of the abaxial epidermis of the seventh fully expanded rosette leaf of Col (A), *nrpb3-1* (B), *tmm-1* (C), *nrpb3-1 tmm-1* (D), *er* *erl1 erl2* (E), *nrpb3-1 er erl1 erl2* (F), *erl1 erl2* (G), *nrpb3-1 erl1 erl2* (H), *er105* *erl2* (I), *nrpb3-1 er105 erl2* (J), *er105* (K) and *nrpb3-1 er105* (L). (M-S) Density of stomata (M,O), stomatal clusters (N,P), paired stomata (Q) and meristemoid-like cells (R,S) on the abaxial surface of the seventh fully expanded rosette leaves. Arrow, paired stomata; bracket, stomatal cluster; arrowhead, meristemoid-like cell. Error bars indicate s.e.m.; **P<0.01 by Student's *t*-test; *n*=30 per genotype. Scale bars: 50 µm.

To investigate the genetic interactions of *NRPB3* with regulators of stomatal patterning, double, triple or quadruple mutants were produced between *nrpb3-1* and *tmm-1*, *er105 erl1 erl2*, *erl1 erl2*, *er105 erl2*, *er105*, *epf2* and *sdd1-1* (Fig. 5 and see Figs S8,S9). The *nrpb3-1 tmm-1* double mutants exhibited dramatically exaggerated *tmm-1* phenotypes. The stomatal density of *nrpb3-1 tmm-1* was significantly higher than that of either the *nrpb3-1* or the *tmm-1* single mutants. Compared with *tmm-1* individual mutants, *nrpb3-1 tmm-1* double mutants not only exhibited larger clusters, but also had a larger number of stomatal clusters of all sizes (Fig. 5B-D,M,N). Similar results were also obtained for *amiR-NRPB3-1 tmm-1* (see Fig. S8). The epidermis of *nrpb3-1 er105 erl1 erl2* quadruple mutants exhibited much higher stomatal density, larger stomatal clusters and an increased number of clustered stomata compared with *er105 erl1 erl2* triple mutants, thus greatly enhancing the *er105 erl1 erl2* phenotypes (Fig. 5E,F,O,P). Furthermore, *nrpb3-1* also exaggerated the stomatal phenotypes of *erl1 erl2*, *er105 erl2* and *er105* (Fig. 5G-L,Q-S). Compared with *nrpb3-1* or *epf2*, many more stomata, paired stomata and meristemoid-like cells were found in *nrpb3-1 epf2* (see Fig. S9C,D,G-I). In addition, surges in both the stomatal density and the number of clustered stomata were observed in *nrpb3-1 sdd1-1* (see Fig. S9E,F,J,K). Overall, *NRPB3* interacted synergistically with these genes in regulating stomatal patterning.

### *NRPB3* genetically interacts with *FAMA*, *FLP*, *ICE1* and *MUTE* in restraining stomatal lineage cell divisions

**Fig. 6.**
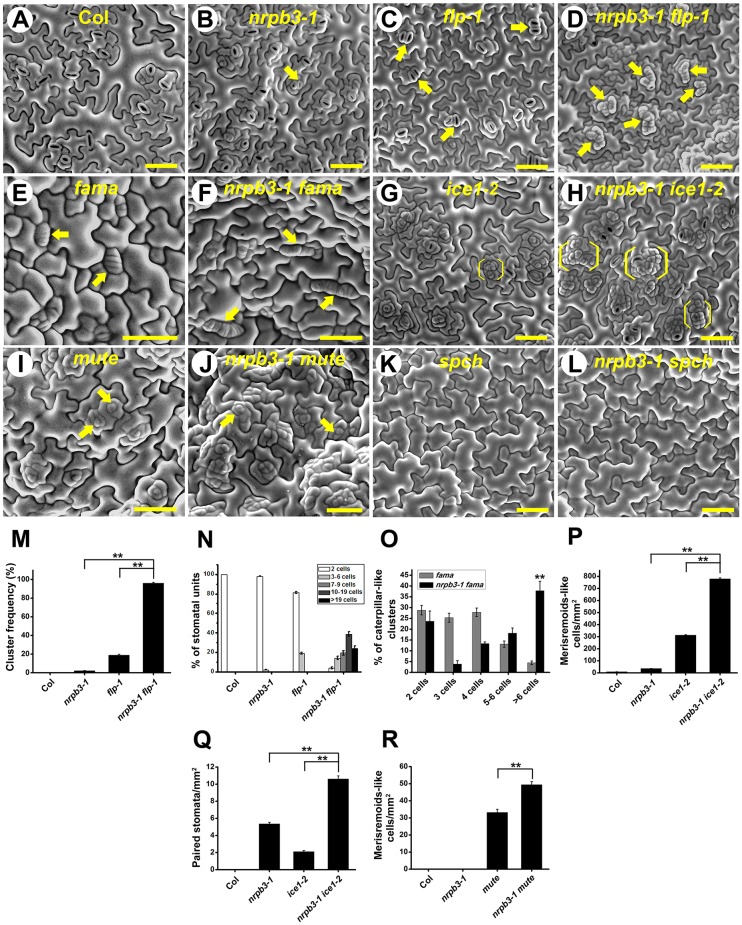
**Genetic interaction analysis between *NRPB3* and stomatal differentiation genes.** (A-D,G,H) SEM images of abaxial epidermis of the seventh fully expanded rosette leaf of Col (A), *nrpb3-1* (B), *flp-1* (C), *nrpb3-1 flp-1* (D), *ice1-2* (G) and *nrpb3-1 ice1-2* (H). (E,F,I-L) SEM images of the abaxial epidermis of 2-week-old cotyledons of *fama* (E), *nrpb3-1 fama* (F), *mute* (I), *nrpb3-1 mute* (J), *spch* (K) and *nrpb3-1 spch* (L). (M) Frequency of clusters per area. (N) The relative means of cells per cluster and of normal stomata in each genotype. (O) The relative means of cells per cluster in each genotype. (P,Q) Density of meristemoid-like cells (P) and paired stomata (Q) on the abaxial surface of the seventh fully expanded rosette leaves. (R) Density of meristemoid-like cells on the abaxial surface of mature cotyledons. Arrows indicate paired stomata (B,C), stomatal clusters (D), caterpillar-like structures (E,F) and meristemoid-like cell (I,J); bracket indicates meristemoid-like cell clusters in G,H. Error bars indicate s.e.m.; **P<0.01 by Student's *t*-test; *n*=30 per genotype. Scale bars: 50 µm.

The molecular character of *NRPB3* led us to investigate its genetic interactions with transcription factors, including *FLP*, *MYB88*, *FAMA*, *ICE1*, *SCRM2*, *MUTE* and *SPCH* (Fig. 6 and see Figs. S10,S11). The *flp-1* mutants typically had two laterally aligned stomata. Severe phenotypes with a larger size and greater frequency of clusters were observed in *nrpb3-1 flp-1* and *amiR-NRPB3-1 flp-1* (Fig. 6C,D,M,N and see Fig. S10). In *fama*, caterpillar-like structures were produced in the normal positions of stomata. Those structures were larger in *nrpb3-1 fama*, strongly exaggerating the phenotype of *fama* (Fig. 6E,F,O). In *nrpb3-1 ice1-2*, larger clusters of meristemoid-like cells were evident, and the number of meristemoid-like cells and paired stomata increased dramatically (Fig. 6G,H,P,Q). Neither *myb88* (SALK_068691) nor *scrm2-1* exhibited any visible defects in stomatal development. The phenotypes of *nrpb3-1 myb88* and *nrpb3-1 scrm2-1* were similar to *nrpb3-1* (see Fig. S11). In *nrpb3-1 mute*, a higher density of undifferentiated meristemoid-like cells was observed (Fig. 6I,J,R). In *nrpb3-1 spch*, the epidermis was only composed of pavement cells and no stomatal lineage was initiated (Fig. 6K,L), suggesting that the involvement of *NRPB3* in stomatal development is dependent on *SPCH*. In summary, *spch*, *mute*, *fama* and *ice1* were epistatic to *nrpb3-1* with regard to stomatal differentiation. Evidently, *NRPB3* genetically interacts with *FAMA*, *FLP*, *ICE1* and *MUTE* in restraining stomatal lineage cell divisions.

### NRPB3 physically interacts with FAMA and ICE1

**Fig. 7.**
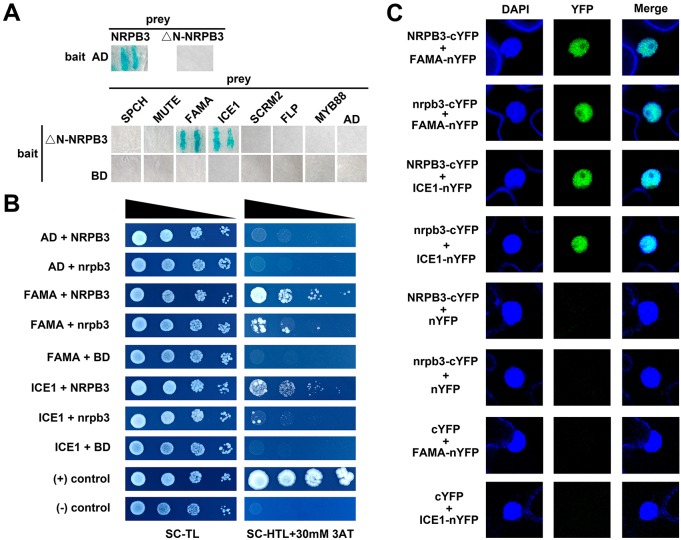
**NRPB3 associates with FAMA and ICE1.** (A) Yeast two-hybrid analysis. Blue indicates reporter activation. (B) The interactions of nrpb3 with FAMA and ICE1 were weaker than those of NRPB3 with FAMA and ICE1. (C) Confocal images of the BiFC analysis. Full-length NRPB3 and nrpb3 were used in B and C. BD, binding domain; AD, activation domain; SC-TL, synthetic complete medium lacking tryptophan and leucine; SC-HTL, synthetic complete medium lacking histidine, tryptophan and leucine; 3AT, 3-amino-1,2,4-triazole.

The genetic interactions between *NRPB3* and the transcription factors involved in stomatal development led us to investigate their interactions at the molecular level. The yeast two-hybrid (Y2H) system was initially used. When NRPB3 was fused with the Gal4 DNA binding domain (BD), transcriptional activation itself was detected. However, it disappeared when the N-terminal 67 amino acids of NRPB3 were deleted (Fig. 7A). The results showed that NRPB3 strongly interacted with FAMA and ICE1, but not interacted with FLP, MYB88, SCRM2, MUTE or SPCH (Fig. 7A). In addition, FAMA was also identified in a Y2H screen, further demonstrating the interactions between NRPB3 and FAMA. In agreement with the Y2H results, functional associations of NRPB3 with FAMA and ICE1 were detected in bimolecular fluorescent complementation (BiFC) assays (Fig. 7C). These results indicated that NRPB3 physically interacts with FAMA and ICE1, both *in vitro* and *in planta*.

To investigate whether the mutation in *nrpb3-1* influenced the physical interactions of NRPB3 with FAMA and ICE1, another Y2H system in which protein-protein binding capability could be measured with yeast growth was used (Fig. 7B and see Fig. S12A). Remarkably, binding affinities of NRPB3 with both FAMA and ICE1 were decreased for the mutation (Fig. 7B), suggesting that this site (residue 172) is crucial for their physical interactions. Clear interactions of nrpb3 with both FAMA and ICE1 were detected in the BiFC system (Fig. 7C). However, it was difficult to conclude whether the mutation influenced their binding abilities in this system, because multiple factors affected the reconstitution of complementary YFP molecules (Lalonde et al., 2008). Additionally, *CYCLIN-DEPENDENT KINASE B1;1* (*CDKB1;1*), which is directly repressed by *FAMA* (Ohashi-Ito and Bergmann, 2006; Hachez et al., 2011), was upregulated in *nrpb3-1* (see Fig. S13), suggesting that the suppression of FAMA on its target gene *CDKB1;1* was impaired by the NRPB3 mutation.

Previous studies revealed that the RETINOBLASTOMA RELATED (RBR) protein represses entry asymmetric cell divisions by binding directly to the *SPCH* promoter and ensures irreversible stomatal terminal differentiation by interacting with FLP, MYB88 and FAMA (Borghi et al., 2010; Weimer et al., 2012; Lee et al., 2014; Matos et al., 2014). This led us to investigate whether RBR was a potential candidate for connecting stomatal signals to Pol II via NRPB3. However, direct interactions between these proteins were not detected in the Y2H system (see Fig. S12B).

### *NRPB3* works together with *FAMA*, *ICE1* and *FLP/MYB88* to limit GMC division during terminal GC differentiation

**Fig. 8.**
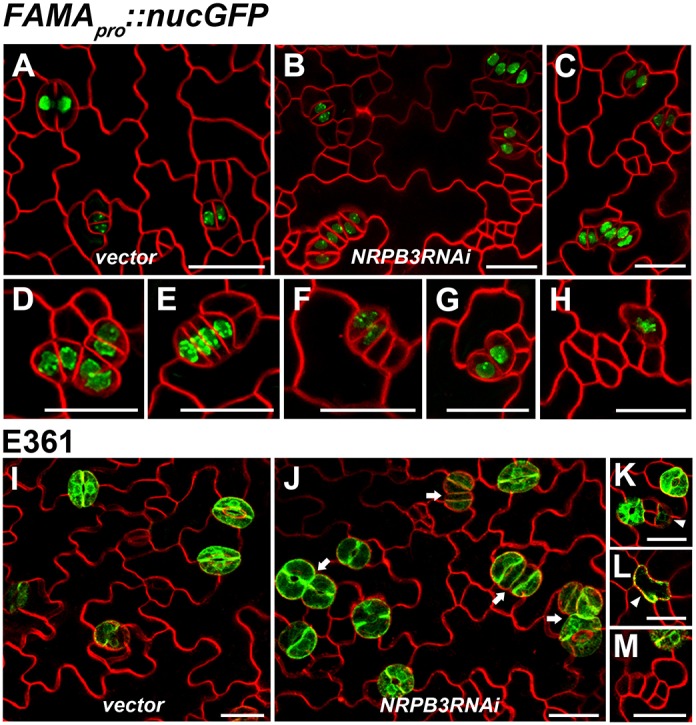
**Expression of *FAMA_pro_::nucGFP* and E361 in *GVG-NRPB3RNAi* transgenic plants.** (A-H) Expression of *FAMA_pro_::nucGFP* in the abaxial epidermis of the sixth immature rosette leaf of vector control (A) and *GVG-NRPB3RNAi* transgenic plants (B-H). (C-H) Close-up of parallel-aligned stomata (C), caterpillar-like structures (D-F) and aberrant GMCs or GCs (G,H) expressing *FAMA_pro_::nucGFP* in *GVG-NRPB3RNAi* transgenic plants. (I-M) Expression of E361 in the abaxial epidermis of the sixth immature rosette leaf of vector control (I) and *GVG-NRPB3RNAi* transgenic plants (J-M). (K,L) Close-up of unpaired GCs expressing E361 in *GVG-NRPB3RNAi* transgenic plants. (M) Close-up of caterpillar-like structures exhibiting no E361 expression in *GVG-NRPB3RNAi* transgenic plants. Arrow, clustered stomata; arrowhead, unpaired guard cell. Scale bars: 20 µm.

**Fig. 9.**
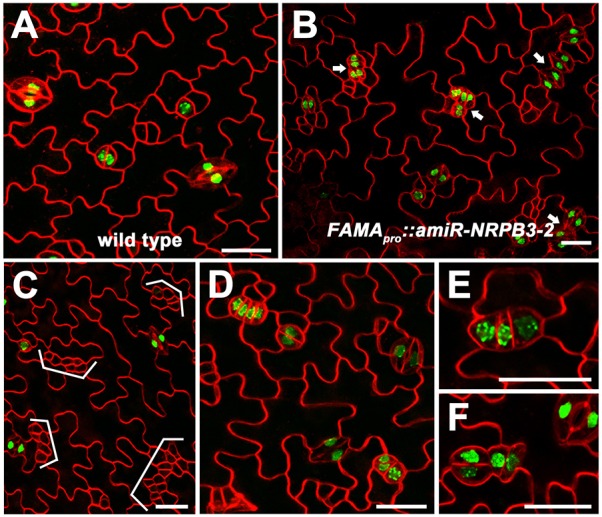
**Expression of *FAMA_pro_::nucGFP* in *FAMA_pro_::amiR-NRPB3-2* transgenic plants.** (A-F) Expression of *FAMA_pro_::nucGFP* in the abaxial epidermis of the sixth immature rosette leaf of the wild type (A) and *FAMA_pro_::amiR-NRPB3-2* transgenic plants (B-F). (C) Close-up of clusters of small, highly divided meristemoid-like cells in *FAMA_pro_::amiR-NRPB3-2* transgenic plants. (D-F) Close-up of caterpillar-like structures (D,E) and aberrant GMCs or GCs (F) expressing *FAMA_pro_::nucGFP* in *FAMA_pro_::amiR-NRPB3-2* transgenic plants. Arrow, clustered stomata; bracket, clusters of small, highly divided meristemoid-like cells. Scale bars: 20 µm.

To confirm the molecular interactions of NRPB3 with FAMA and ICE1, the stomatal cell-specific markers *FAMA*, which marks GMCs and GCs (Ohashi-Ito and Bergmann, 2006), and E361, which marks mature GCs (Gardner et al., 2009), were used to determine the cell identity in caterpillar-like structures in *nrpb3* mutants. In *GVG-NRPB3RNAi* and *amiR-NRPB3-1* plants transformed with *FAMA_pro_::nucGFP*, strong GFP signals were observed in the parallel-aligned stomata and caterpillar-like structures (Fig. 8A-F and see Fig. S14A-D). In addition, aberrant GMCs or GCs were occasionally observed (Fig. 8G,H and see Fig. S14E). In *GVG-NRPB3RNAi* plants marked with E361, GFP signals could be observed in clustered stomata and unpaired guard cells (Fig. 8I-L), but not in caterpillar-like structures (Fig. 8M). These results indicated that the same caterpillar-like structures as those in *fama* or *ice1* were produced in *nrpb3* mutants. The function of *NRPB3* in terminal GC differentiation was further investigated using *amiR-NRPB3-2* driven by the *FAMA* promoter (*FAMA_pro_::amiR-NRPB3-2*). The *FAMA_pro_::amiR-NRPB3-2* construct induced clusters of stomata and small, highly divided meristemoid-like cells and dramatically exaggerated the *flp-1* phenotype (Fig. 9A-C and see Fig. S15). More importantly, caterpillar-like structures expressing *FAMA_pro_::nucGFP* were observed in *FAMA_pro_::amiR-NRPB3-2* plants (Fig. 9D-F). Altogether, these results suggest that *NRPB3* works together with *FAMA*, *ICE1* and *FLP/MYB88* to limit GMC division during terminal GC differentiation.

### NRPB2, the second largest subunit of Pol II, is also required for stomatal development

The requirement of *NRPB3* for stomatal development indicates that functional Pol II might be crucial for this process. All of the null mutants of Pol II genes identified to date are lethal (Onodera et al., 2008; Ream et al., 2009). However, a weak allele of the second largest subunit of Pol II (*NRPB2*) has been isolated as *nrpb2-3* (Zheng et al., 2009). Increased stomatal cell density and paired stomata were observed in *nrpb2-3* and *nrpb3-1 nrpb2-3* (see Fig. S16A-D,I,J). Additionally, *nrpb2-3* dramatically enhanced the phenotypes of both *tmm-1* and *flp-1* (see Fig. S16E-H,K-N). These results indicate that NRPB2 is involved in stomatal patterning and differentiation. Taken together, we concluded that Pol II plays an essential role in stomatal development.

## DISCUSSION

The partial loss-of-function mutants of *NRPB3* exhibit pleiotropic phenotypes and its homozygous T-DNA mutants are lethal, indicating that functional NRPB3 is essential for plant viability and development. *NRPB3* is strongly expressed in the tissues and cells that show high mitotic activity, suggesting that its function is closely related to cell division. Furthermore, a much higher number of both stomatal and non-stomatal cells were produced upon its mutation, indicating that NRPB3 largely affects cell division and cell cycle regulators may be its targets.

Developmental signals are transmitted to Pol II, regulating the transcription of target genes. Thus, the mutation of NRPB3 could cause widespread effects on the stomatal signaling pathway. Consistent with this view, the expression of several stomatal development genes was indeed changed in *nrpb3* mutants and severe stomatal development defects were observed. In this sense, it is not surprising that *NRPB3* synergistically interacts with the known stomatal regulators genetically. It has been reported that several factors, such as plasmodesmatal permeability, sterols, auxin transport and the microRNA pathway, regulate stomatal development in parallel to the TMM-MAPK signaling pathway (Kutter et al., 2007; Guseman et al., 2010; Kong et al., 2012; Qian et al., 2013; Le et al., 2014; Yang et al., 2014). Therefore, we cannot exclude the possibility that *NRPB3* regulates another independent pathway in stomatal development.

Pol II receives genetic regulatory information from tens of thousands of sequence-specific DNA binding transcription factors (Kadonaga, 2004). Signal transmission from these transcription factors to Pol II is extremely complicated. During this process, the multisubunit Mediator complex, which is broadly required for transcription by Pol II, bridges between gene-specific transcription factors and the general Pol II machinery (Conaway and Conaway, 2011; Larivière et al., 2012). It can directly integrate inputs from multiple signal-regulated transcription factors through its specialized subunits, recruit Pol II to target promoters and regulate the assembly of the Pol II initiation complex (Carrera and Treisman, 2008; Conaway and Conaway, 2011). Previous research has found that the Pol II subunit RPB3 directly interacts with the Mediator subunit Med17 and mutations in RPB3 (C92R, A159G) affect global Pol II recruitment and transcription *in vivo* (Soutourina et al., 2011). Therefore, the interactions between Pol II and the Mediator in *nrpb3* mutants might be influenced. Thus, Pol II recruitment to the target promoter could be disturbed, interfering with the signals regulated by transcription factors. Some signal-regulated transcription factors can directly interact with RPB3. In bacteria, several lines of evidence show that transcriptional activation by the catabolite gene activator protein (CAP) involves its direct interaction with the RNA polymerase α subunit, the homolog of RPB3 (Ebright and Busby, 1995). In yeast, two special regions of RPB3, residues 92-95 and 159-162, which are close to each other on the crystallographic structure of Pol II, are considered as an activation target of the transcription activator (see Fig. S17) (Tan et al., 2000). Further research has shown that the region of animal RPB3 that corresponds to residues 92-95 of yeast RPB3 interacts with the transcription factor myogenin during muscle differentiation (Corbi et al., 2002). We found interactions of NRPB3 with the bHLH transcription factors, FAMA and ICE1. Intriguingly, the mutated site G172E in *nrpb3-1* corresponds to residue 162 of yeast RPB3 (see Fig. S17) and this mutation decreased the binding affinities of NRPB3 with both FAMA and ICE1. This finding suggests that RPB3 or its homolog-mediated mechanisms of activation in bacteria, yeast and animals also exist in plants.

**Fig. 10.**
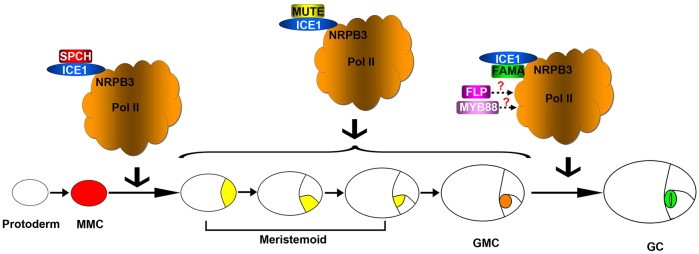
**A proposed model for the function of NRPB3 during stomatal development.** During MMC to meristemoid and meristemoid to GMC transitions, signal transmission from SPCH and MUTE to Pol II might partially depend on their separate interactions with the shared protein ICE1. During terminal GC differentiation, both ICE1 and FAMA could directly transmit their mediated signals to Pol II by associating with NRPB3, whereas signal transmission from FLP/MYB88 to Pol II might rely on unknown proteins.

On the basis of our results, a model for the function of NRPB3 in stomatal development is proposed (Fig. 10). During MMC to meristemoid and meristemoid to GMC transitions, signal transmission from SPCH and MUTE to Pol II might partially depend on their separate interactions with the shared protein ICE1. During terminal GC differentiation, both ICE1 and FAMA could directly transmit their mediated signals to Pol II by associating with NRPB3, whereas signal transmission from FLP/MYB88 to Pol II might rely on unknown proteins (Fig. 10). Therefore, mutation of NRPB3 would disrupt the proper function of these transcription factors, especially that of FAMA and ICE1. Consistent with this view, the *nrpb3* mutants produced caterpillar-like structures similar to those of *fama* or *ice1* and caused large genetic exaggerations of the phenotypes of *flp-1*, *fama*, *ice1* and *mute*. Recent studies have shown that SPCH, together with SCRMs (ICE1/SCRM1 and SCRM2), can directly activate the expression of *TMM*, which in turn inhibits SPCH and SCRMs (Lau et al., 2014; Horst et al., 2015). Thus, the reduced or disrupted binding affinities between NRPB3 and ICE1 in the *nrpb3* mutants might lead to the partial suppression of *TMM* expression, resulting in the delayed degradation of SPCH. This ultimately limits the ability of cells to exit the stomatal lineage, promoting the formation of a great many more stomatal lineage cells. In contrast to *TMM*, *SPCH* expression does not rely on functional *SPCH* or *SCRMs* (Horst et al., 2015). Hence, in the case of *SPCH* upregulation in the *nrpb3* mutants, it is likely that the mutation of NRPB3 disrupts the function of unidentified inhibitors, which could interact with NRPB3 and directly repress *SPCH*. Future work is required to elucidate the underlying mechanisms.

Some parallels between the muscle cell and stomata differentiation are emerging (Pillitteri and Torii, 2007; Serna, 2009; Matos and Bergmann, 2014). Four tissue-speciﬁc bHLH regulators (MyoD, myogenin, Myf5 and MRF4), which are sequentially expressed, function as heterodimers with ubiquitously expressed bHLH factors (E-like proteins) and specify successive cell fate transitional steps in myoblast differentiation (Lassar et al., 1991; Weintraub, 1993). Analogously, three consecutive cell fate transitional steps in stomatal differentiation are directed by three specifically expressed bHLH transcription factors (SPCH, MUTE and FAMA), which also form heterodimers with broadly expressed bHLH-LZ proteins (ICE1 and SCRM2). Similar to RPB3, which is involved in myogenesis by interacting with the bHLH regulator myogenin, NRPB3 participates in stomatal differentiation by associating with the bHLH transcription factors, FAMA and ICE1. This further highlights the surprisingly similar mechanisms for muscle cell and stomata differentiation.

## MATERIALS AND METHODS

### Plant materials and growth conditions

*Arabidopsis thaliana* Col-0 was used as the wild type. The mutants and transgenic lines used in this study were as follows: *tmm-1*, *flp-1*, *er105*, *er105 erl1-2 erl2-1*, *erl1-2 erl2-1*, *er105 erl2-1*, *mute*, *scrm2-1*, *nrpb3-2*, *epf2-1*, *ice1-2*, *myb88* (SALK_068691), *sdd1-1*, *fama-1*, *spch-1*, *nrpb2-3*, *TMM_pro_::TMM-GFP*, *TMM_pro_::nucGFP*, *MUTE_pro_::GFP*, *FAMA_pro_::nucGFP* and E361. Details of sources are provided in supplementary Materials and Methods. All *nrpb3-1* genotypes used for genetic analysis were generated by crossing and were confirmed using the primers listed in Table S1. Seedlings were grown initially on 1/2 MS medium and then transferred to soil in a greenhouse at 20-22°C with 16 h light:8 h dark cycles. The solution contained 20 μM Dex and 0.01% (w/v) Tween-20 was sprayed onto 2-week-old *GVG-NRPB3RNAi* transgenic plants.

### Map-based cloning of *NRPB3*

Plants with the *nrpb3-1* phenotype were isolated as recombinants from F2 plants of a cross between the *nrpb3-1* (Col-0 ecotype) and Landsberg *erecta* (L*er*). Approximately 10,000 F2 plants were used for mapping the *NRPB3* locus. DNA markers that were used for detecting polymorphisms between ecotypes (Col-0 and L*er*) were obtained from an *Arabidopsis* mapping platform (AMP) (Hou et al., 2010). The *nrpb3-1* mutation was mapped to a 110 kb genomic region on chromosome 2. All candidate genes in this region were sequenced and a G/A mutation in *At2g15430* was identiﬁed.

### Imaging and microscopy analysis

Fluorescence of GFP, propidium iodide (PI), 4′,6-diamidino-2-phenylindole (DAPI) and FM4-64 was captured using an Olympus FV1000MPE2 confocal microscope. Scanning electron microscopy (SEM) images were obtained using a Hitachi S-3400N scanning electron microscope. Images were taken from the same region that is in the middle part of leaves and far away from leaf vein. Each SEM image used for analysis represents 0.28 mm^2^. Quantification of epidermal cell types has been described previously (Lai et al., 2005; Balcerowicz et al., 2014).

### GUS staining assays

The approach for GUS staining has been described previously (Qian et al., 2013). The T2 transgenic plants of six independent lines carrying the *NRPB3_pro_::NRPB3-GUS* construct were used for analysis.

### Plasmid construction and generation of transgenic plants

The Gateway cloning system (Invitrogen) was used to construct plasmids as detailed in supplementary Materials and Methods. All the expression constructs were transferred into appropriate *Arabidopsis* plants by the floral dip method (Clough and Bent, 1998).

### Transient expression

Transient expression in *Arabidopsis* protoplasts was performed as described previously (Yoo et al., 2007).

### Real-time PCR analysis

The method used for real-time PCR has been described previously (Qian et al., 2013). For each real-time PCR experiment, at least three biological replicates were conducted. See Table S1 for DNA primer sequences.

### Yeast two-hybrid assay and two-hybrid screen with N-terminally deleted NRPB3

Yeast two-hybrid assay was carried out using the MATCHMAKER two-hybrid system 3 (Clontech) as detailed in the supplementary Materials and Methods. For the yeast two-hybrid screen, yeast strain Y190 transformed with bait pGBK-ΔN-NRPB3 was retransformed with a prey library made from 3-day-old seedlings in pACT (ABRC stock CD4-22) and β-gal activity was assayed according to the manufacturer's protocol (Clontech) as described in more detail in supplementary Materials and Methods.

#### BiFC

Leaves of 3-week-old *Nicotiana benthamiana* were transformed by injection of *Agrobacterium* GV3101 strains containing BiFC constructs (Lavy, 2002) as described in supplementary Materials and Methods. Leaves were incubated with 0.2 mg/l DAPI to stain nuclei and YFP signal was examined 2 days after injection using an Olympus FV1000MPE2 confocal fluorescence microscope. Each interaction was tested at least three times.
